# Crossing-Over in a Hypervariable Species Preferentially Occurs in Regions of High Local Similarity

**DOI:** 10.1093/molbev/msu242

**Published:** 2014-08-18

**Authors:** Vladimir B. Seplyarskiy, Maria D. Logacheva, Aleksey A. Penin, Maria A. Baranova, Evgeny V. Leushkin, Natalia V. Demidenko, Anna V. Klepikova, Fyodor A. Kondrashov, Alexey S. Kondrashov, Timothy Y. James

**Affiliations:** ^1^School of Bioengineering and Bioinformatics, Moscow State University, Moscow, Russia; ^2^Institute of Information Transmission Problems, Russian Academy of Sciences, Moscow, Russia; ^3^Department of Biology, Lomonosov Moscow State University, Moscow, Moscow, Russia; ^4^Bioinformatics and Genomics Programme, Centre for Genomic Regulation (CRG) Barcelona, Spain; ^5^Universitat Pompeu Fabra (UPF), Barcelona, Spain; ^6^Institució Catalana de Recerca i Estudis Avançats (ICREA), 23 Pg. Lluís Companys, Barcelona, Spain; ^7^Department of Ecology and Evolutionary Biology, University of Michigan, Ann Arbor, MI

**Keywords:** *S. commune*, recombination, negative selection, hyperpolymorphic

## Abstract

Recombination between double-stranded DNA molecules is a key genetic process which occurs in a wide variety of organisms. Usually, crossing-over (CO) occurs during meiosis between genotypes with 98.0–99.9% sequence identity, because within-population nucleotide diversity only rarely exceeds 2%. However, some species are hypervariable and it is unclear how CO can occur between genotypes with less than 90% sequence identity. Here, we study CO in *Schizophyllum commune*, a hypervariable cosmopolitan basidiomycete mushroom, a frequently encountered decayer of woody substrates. We crossed two haploid individuals, from the United States and from Russia, and obtained genome sequences for their 17 offspring. The average genetic distance between the parents was 14%, making it possible to study CO at very high resolution. We found reduced levels of linkage disequilibrium between loci flanking the CO sites indicating that they are mostly confined to hotspots of recombination. Furthermore, CO events preferentially occurred in regions under stronger negative selection, in particular within exons that showed reduced levels of nucleotide diversity. Apparently, in hypervariable species CO must avoid regions of higher divergence between the recombining genomes due to limitations imposed by the mismatch repair system, with regions under strong negative selection providing the opportunity for recombination. These patterns are opposite to those observed in a number of less variable species indicating that population genomics of hypervariable species may reveal novel biological phenomena.

## Introduction

Recombination between double-stranded DNA molecules is a key genetic process which occurs in a wide variety of manners in all kinds of organisms. In particular, reciprocal meiotic recombination, which involves crossing over (CO) between homologous chromosomes, is an indispensible part of sexual reproduction, although sometimes CO is limited to one sex only (Burt et al. 1991). Usually, CO occurs between genotypes that have 98.0–99.9% sequence similarity to each other, because within-population nucleotide diversity only rarely exceeds 2% (Leffler et al. 2012). However, some species are hypervariable (Cutter et al. 2013; Dey et al. 2013) and sexual reproduction within a hypervariable population must involve CO between much more dissimilar genotypes.

There are at least two reasons why studying CO between genetically distant genotypes is interesting. First, interparental nucleotide diversity strongly affects our ability to precisely map recombination events and estimate linkage. Three methods can be used to estimate the locations of CO events and determine how they are effected by the genomic environment: Parent-offspring and parent-gamete comparison (Mancera et al. 2008; Kong et al. 2010; Roach et al. 2010; Wang et al. 2012) DNA double strand break (DSB) mapping (Pan et al. 2011; Smagulova et al. 2011) and LD analysis (International HapMap 2005; Myers et al. 2005; Chan et al. 2012). Obviously, the scale at which cross overs can be resolved using parent-offspring comparisons is limited by the density of differences between the recombining genotypes, because a CO event can be localized only to an interval between two consecutive differences.

Second, differences between recombining chromosomes are known to interfere with CO and may encourage nonreciprocal, ectopic recombination. Recombination between divergent homologues may not occur due to two possible reasons: It could be rarely initiated, because of the low density of regions of perfect identity of sufficient length (Shen and Huang 1986; Datta et al. 1997), or due to delocalized interference by the mismatch repair (MMR) system (Borts and Haber 1987; Waldman and Liskay 1988; Datta et al. 1997; Opperman et al. 2004; Emmanuel et al. 2006). Sequence divergence between mitotically recombining DNAs strongly affects CO in yeast, where 14% nt divergence decreases the rate by a factor of 330 (Datta et al. 1997). Data on experimental constructs show that in mammals 19% divergence essentially precludes recombination (Waldman and Liskay 1988), and even adding a few single nucleotide polymorphisms (SNPs) that shorten regions of perfect identity between two DNA sequences from 232 to 134 nt reduced the recombination rate between them by a factor of 20. A similar effect of chromosome-scale sequence divergence has been observed in *Arabidopsis thaliana* in which the CO rate within a reporter construct in isogenic lines was 1.5 times higher than in outbred lines (Emmanuel et al. 2006). Knockouts of genes involved in the MMR pathway increase the rate of recombination, especially between divergent sequences (Datta et al. 1997; Opperman et al. 2004; Emmanuel et al. 2006). Recombination between sequences highly diverged at the chromosome level may also cause meiotic death. *Saccharomyces cerevisiae* that has one copy of chromosome III introgressed from *S*. *paradoxus* possesses a reduced recombination rate and an increased spore mortality due to nondisjunction in meiosis I (Chambers et al. 1996).

Sensitivity of the mechanisms of recombination to the degree of similarity between recombining sequences may have evolved because recombination between paralogous genome regions causes chromosome rearrangements (Sasaki et al. 2010; Lichten and Massy 2011). Thus, one might expect CO to be rare in hypervariable species. Alternatively, mechanisms of CO in such species may be rather different from those in species with ordinary levels of nucleotide diversity.

*Schizophyllum commune* is a hypervariable species, with DNA polymorphism values (average pairwise dissimilarity) of *π* = 0.06 and *π* = 0.05 within the US and European populations, respectively (Baranova MA et al., in preparation). Although the genetic distance between these two populations is 0.14, US and European individuals can be freely crossed and produce viable offspring (Raper and Miles 1958). Other advantages of using *S*. *commune* are its small genome of only 38.5 Mb (Ohm et al. 2011) and the ease at which the fungus can be cultivated in both the haploid and dikaryotic (genetically diploid) phases. Sequencing of haploid individuals greatly simplifies genome assembly and abolishes the need for SNP phasing.

These features make *S*. *commune* a suitable model organism for studying recombination and its dependence on genetic differences between the recombining sequences.

## Results

We sequenced genotypes of two haploid *S*. *commune* individuals, each isolated from a single meiotic spore from fruiting bodies collected in Russia (Moscow) and in the United States (The Everglades, FL), together with genotypes of 17 of their F_1_ haploid offspring, with coverage from 33 to 124 (supplementary table S1, Supplementary Material online). We used randomly isolated meiotic spores as opposed to tetrad analysis, and therefore we focus on reciprocal recombination rather than gene conversion. In basidiomycota, it is very hard to collect tetrads of spores produced in individual meiosis, and thus we could not consider the consequence of the same CO in two offspring genotypes. In contrast to other studies of recombination which used alignments of reads to reference assemblies (Mancera et al. 2008; Roach et al. 2010; Wang et al. 2012), we assembled reads into scaffolds independently for each genotype. The N50 for all genotypes exceeded 40 kb (supplementary table S1, Supplementary Material online). To investigate linkage disequilibrium (LD) and perform population analysis, we also sequenced 13 individuals from the United States and 19 individuals from European Russia (Baranova MA et al., in preparation), and all individuals have N50 >3 kb and coverage >30 × (supplementary table S2, Supplementary Material online). We prepared a multiple alignment of 17 F_1_ offspring, parental genotypes from Moscow and Ann Arbor and the reference genome of *S*. *commune* (Ohm et al. 2011) using MultiZ (Blanchette et al. 2004). Then, we identified all cases within alignments where an offspring genotype switched identity from one parental genotype to another along the alignment. For population data, we also performed multiple alignments and included the reference genome to order scaffolds of other individuals.

**Fig. 1.**
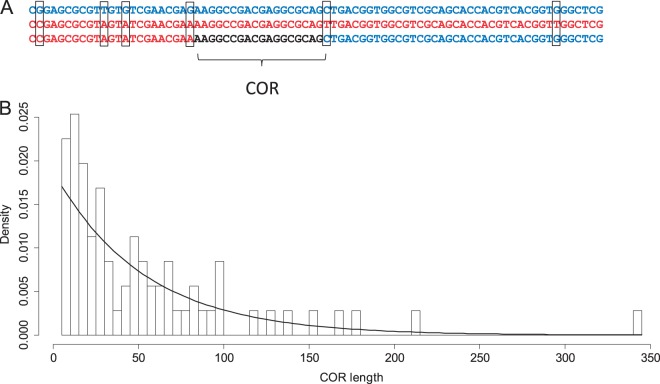
COR description and distribution of CORs lengths. (*A*) alignment of parental and offspring alleles. Moscow parent shown in blue and the Everglades parent shown in red. COR shown in black. (*B*) Distribution of CORs lengths, curve is an exponential fit.

**Fig. 2.**
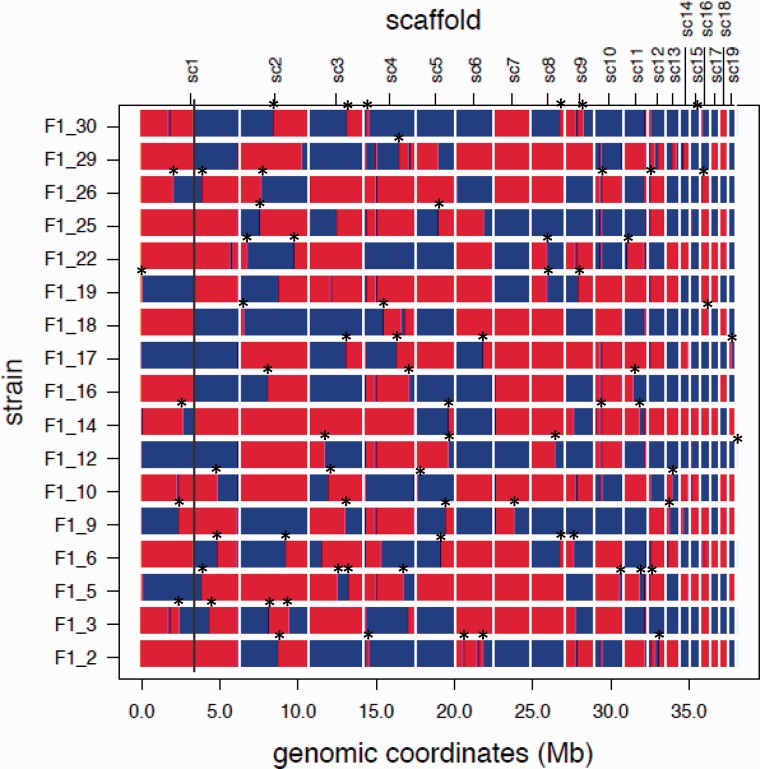
Offspring genotypes and observed COs. Each line corresponds to offspring genotypes, region matched with the Everglades parent is shown in red, and region matched with Moscow parent is shown in blue. Line shows example of rearrangement or misalignment in reference genome, where 7 out of 17 offspring have a template switch, but there are no reads confirm COs in this region. The genotype for each region was determined using a sliding window approach with windows of size 100,000 bp and a step size of 25,000 bp. Seventy-one crossovers, which passed filtration, are shown with an asterisk.

We defined a crossover region (COR) as a segment of an F_1_ offspring genotype that is flanked by two successive interparental differences, each occupied by different nucleotides (alleles) in the two parents (fig. 1*A*). Therefore, COR length is the length of an identity tract between parental genotypes where CO occurs. Obviously, CORs tend to be shorter in genome regions with higher nucleotide diversity. Because of possible errors in the alignment, such as alignment of paralogous instead of orthologous regions, the assembly and the alignment of each COR were rigorously tested, and a conservative set of 71 definite CO events within the 17 F_1_ genotypes was obtained (fig. 2, supplementary table S3 and data S1, Supplementary Material online). We confirmed the accuracy of our results by Sanger resequencing of 24 randomly chosen DNA segments of F_1_ genotypes, each containing a COR (supplementary table S4, Supplementary Material online).

To avoid false CO detection, we excluded double crossovers that transferred less than 10, 50, or 100 SNPs and obtained the same set of 71 COs for all these thresholds. Among these COs, we found one event in which the offspring genotype displayed a “Moscow-Florida-Moscow-Florida” pattern (supplementary fig. S1, Supplementary Material online), which is likely to be complex conversion tract (Duret and Galtier 2009).

### COs Are Associated with Regions of High Local Similarity

Because the genetic distance between the two parental genotypes is 0.14, the average distance between the two successive interparental nucleotide differences is only 7 nt (9 nt within exons). The expected length of a COR, if COs and interparental differences were distributed randomly, is two times higher than the average distance between interparental differences, because of the properties of the exponential distribution. However, the average observed COR length is 53 nt (median = 31), almost four times higher than the expected 14 nt. Thus, our data clearly demonstrate that COs preferentially occur within regions of higher interparental similarity (fig. 1*B*), in line with the results showing obstacles to recombination between divergent sequences (Datta et al. 1997; Opperman et al. 2004; Emmanuel et al. 2006). Lengths of individual CORs vary substantially, with 6 out of 71 CORs being shorter than 10 nt, and the distribution of CORs lengths is not different from exponential *P* value = 0.20, Kolmogorov–Smirnov test).

### Selection Is the Source for High Local Identity Associated with COs

**Fig. 3.**
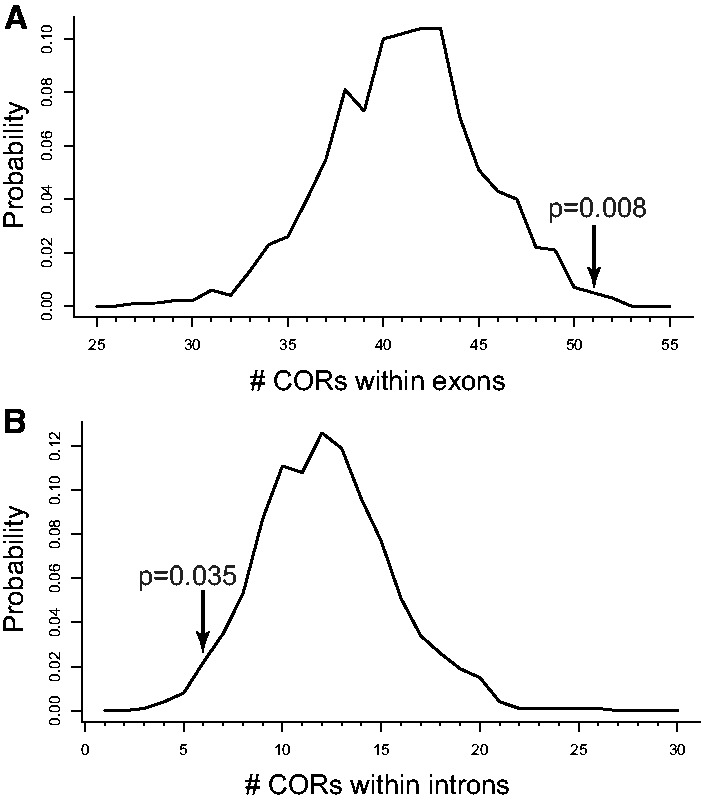
CORs prefer exons. Curves show expected numbers of CORs which (*A*) reside within exons or (*B*) reside within introns. The distributions based on 1,000 permutations for genome regions with the same identity tract lengths. Arrows correspond to the observed values.

Higher sequence similarity between the two parental genotypes around sites of CO could be due to locally stronger negative selection. To investigate whether negative selection affects CORs, we looked at the genome annotation and polymorphism data. Using the annotation from Ohm et al. (2011), we identified a strong association between CORs and exons. Fifty-one CORs reside within exons, which is higher than randomly expected (*P* value = 0.008, permutation test), but CORs are underrepresented within introns (6 cases, *P* value = 0.035, permutation test) (fig. 3). Thus, CORs are associated not just with open chromatin as in yeasts (Berchowitz et al. 2009) or transcribed regions, but most specifically with the more conserved exon sequences. Still, the average COR length is three times higher than expected for randomly chosen exons.

**Fig. 4.**
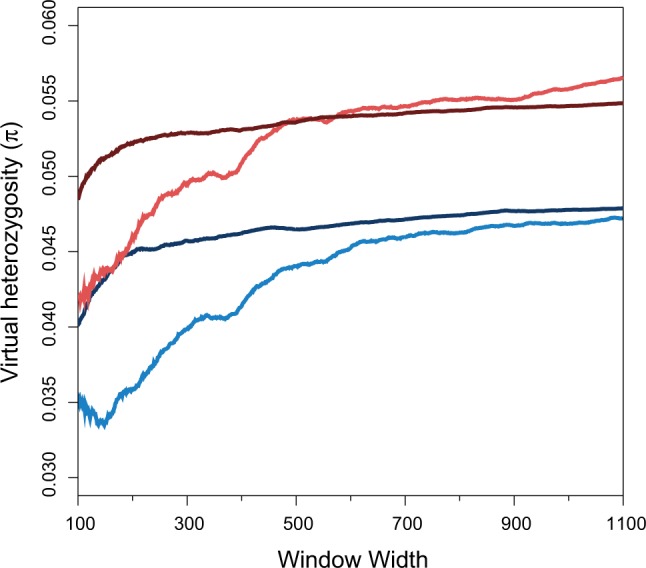
Nucleotide diversity (*π*) near CORs. Light blue and light red curves show observed π in windows of different widths centred at the middle of CORs for Russian and US populations; dark blue and dark red curves shows π in windows centered at bootstrapped regions with the same lengths of identity to observed CORs and the same exon enrichment, but not associated with an observed CO (see Materials and Methods).

We also observed that COs in our study preferentially occurred within regions that also demonstrated reduced genetic variation within both US and Russian populations of *S*. *commune*. This pattern could be solely due to a local correlation between the within-population variation and the divergence of the two parental genotypes. To test this explanation, we performed a Monte-Carlo test by sampling genome regions with the same distance between parental nucleotide differences as the CORs lengths and with the same exon enrichment, but not associated with observed COs. We found that the reduction of within-population variation was much more pronounced around authentic CORs than around simulated CORs (fig. 4). Therefore, low within-population variation around CORs is mostly due to negative selection preserved in both populations.

**Fig. 5.**
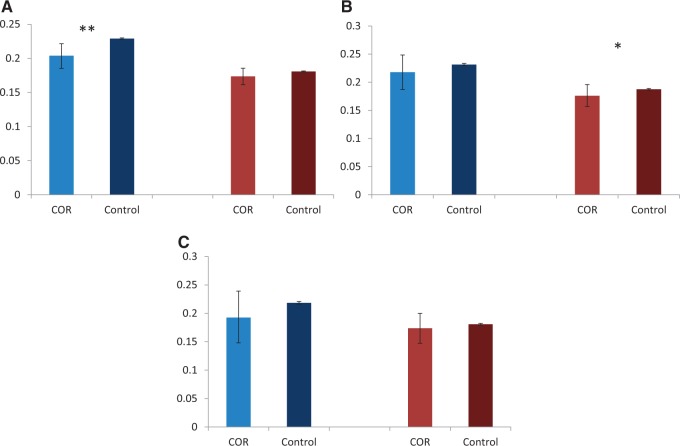
MAF in CORs. MAFs in ten randomly sampled individuals from the Russian population (blue bars) and the US population (red bars). MAFs were recorded for polymorphic loci within CORs and within 10-kb regions centred at CORs for control. (*A*) All sites, (*B*) synonymous sites, and (*C*) nonsynonymous sites. *and **correspond to *P* values < 0.05 and < 0.01, respectively (Mann–Whitney *U* test). Five percent confidence intervals obtained from 1,000 permutations.

Minor allele frequencies (MAFs) at polymorphic sites within CORs are reduced (fig. 5). This reduction is significant for all sites in the Russian population (*P* value = 0.01, Mann–Whitney *U* test) and for synonymous sites in the US population (*P* value = 0.02, Mann–Whitney *U* test) this results concordant with lower values of Tajima’s *D*-statistics in CORs for this comparisons (supplementary table S5, Supplementary Material online). This observation is also consistent with the action of negative selection.

### COs Are Associated with Elevated GC-Content, due to Exon Enrichment

**Fig. 6.**
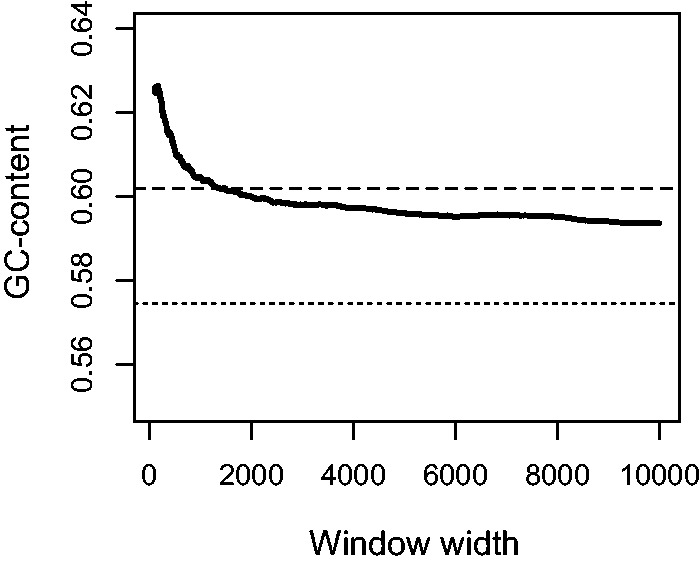
GC-content for windows of different widths centered in a COR. Dotted line shows the average GC-content genome-wide and dashed line shows the average GC-content for exons.

In *S*. *commune* COs are associated with a higher than genome average GC-content (62% for CORs vs. 58% genome average, χ^2^ *P* value = 0.03) (fig. 6) in line with the results for the majority of species in which this correlation has been studied (Birdsell 2002; Kong et al. 2002; Kim et al. 2007; Backström et al. 2010; Smagulova et al. 2011). In *S*. *commune* exons are GC-rich and GC-content in CORs does not significantly differ from that in exons (62% vs. 60%, χ^2^ *P* value = 0.38). Therefore, increased GC-content in CORs can be solely explained by their co-occurrence with exons.

### (CNN)n Motif Overrepresented in CORs

To study specific sequence patterns associated with Cos, we looked for evidence of their motif enrichment using MEME (Bailey and Elkan 1994). We performed two comparisons: CORs versus their immediate flanks and CORs together with 400-nt flanks versus adjacent regions of the same length. In the first comparison, we did not find any significant motifs, possibly due to the shortness of the considered sequences and the small set of only 71 events. In the second comparison, a CCNCNNCNNCNNCNNCNNCNN (*e* value 8.2 × 10^−^^42^) motif was associated with COs. The observed motif remains significant when CORs were compared with a set of random exon sequences (*e* value 9.9 × 10^−^^34^).

### COs Predict Decreased LD

**Fig. 7.**
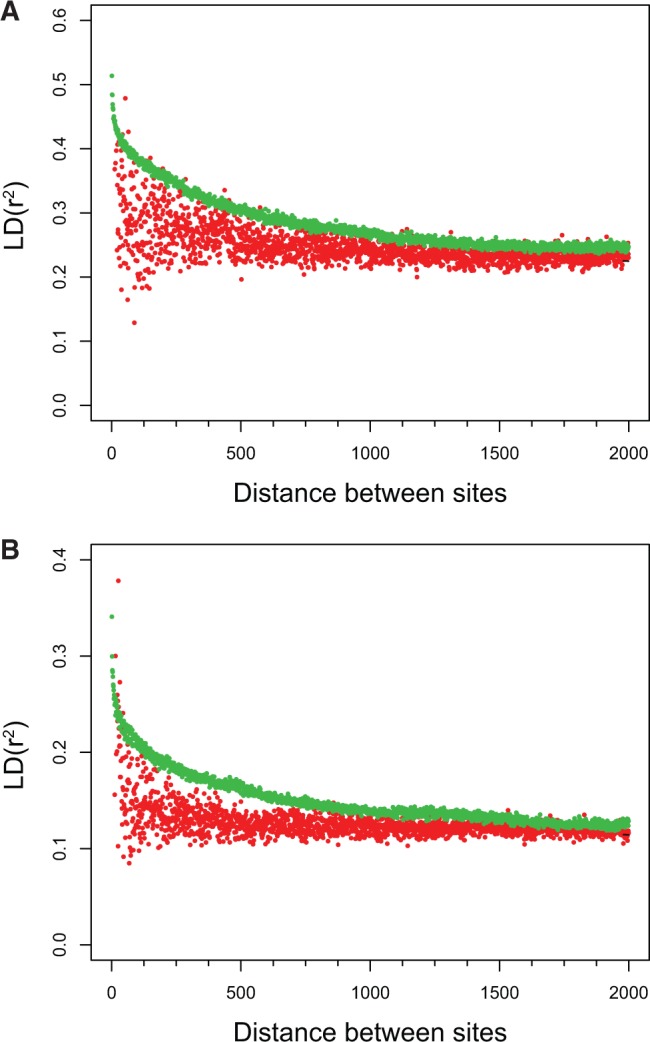
Low LD near COs. Values of LD for (*A*) ten Russian individuals and (*B*) for ten US individuals. Red dots correspond to pairs of SNPs separated by the middle of a COR, and green dots correspond to pairs of SNPs located 10,000 nt away from a COR. Parental sequences were not included in these analyses.

In both the US and the Russian populations, LD between pairs of SNPs at opposite sides of a CO is markedly reduced (fig. 7). Indeed, LD for close SNPs not separated by CO is high, and disappears only when the distance between such SNPs exceeds ≈1,000 nt. By contrast, LD is low even for very close SNPs separated by a CO. This effect indicates that recombination is frequent between most SNPs associated with COs and, thus, that COs mostly occurred at recombination hotspots. Higher LD in the Russian population, together with its lower nucleotide diversity, indicates that this population has a lower population effective size than the US population, consistent with earlier estimates of global population structure and diversity (James et al. 2001).

## Discussion

Genetic distances of ≈0.1 are often associated with a substantial, or even complete, reproductive isolation (Mendelson et al. 2004; Elliot and Crespi 2006). However, the two parents used in our study produced viable, phenotypically normal offspring, despite having 0.14 of their nucleotides different. Thus, *S*. *commune* is an excellent system for studying fine-scale recombination between genotypes that are one or even two orders of magnitude more distant from each other than that which can be achieved in *S*. *cerevisiae* (Mancera et al. 2008), *Drosophila melanogaster* (Begun et al. 2007), *A*. *thaliana* (Nordborg et al. 2005), or *Homo sapiens* (MacArthur et al. 2012). A very high density of interparental differences provides a unique opportunity to localize CO events to CORs of lengths << 100 nt.

### Divergence Interferes with Recombination

We identified 71 CO events among 17 offspring (fig. 2 and supplementary table S3, Supplementary Material online), which is lower than what would be expected (11*17 = 187) if at least one CO event occurred per chromosome (Lynn et al. 2004). This discrepancy can be an artifact of rigorous filtering of our data, because we may have discarded some bona fide events, in particular, those located near scaffold boundaries, while trying to avoid false events. Alternatively, our observations may reflect a real reduction of the CO rate in the interpopulation cross we studied, due to a high genetic distance between the parents (Waldman and Liskay 1988; Datta et al. 1997). In particular, inversion differences between the parental genotypes may interfere with recombination, and we detected 294 such inversions using MUMer (Kurtz et al. 2004) (data not reported). We did not validate any putative COs that lay on scaffold borders. To control for biases that might be introduced by filtering, we repeated the analysis after excluding 500 nt at each scaffold boundary. This exclusion had almost no effect on nucleotide diversity and exon content.

The association of COs with regions of higher similarity between the parental genotypes (fig. 1*B*) is likely caused by obstacles to CO between divergent homologues. These obstacles can arise if there is a minimal length of perfect identity tract needed for initiation of recombination (MEPS, minimal efficient processing segment, Shen and Huang 1986), and/or if MMR aborts recombination which involves formation of divergent heteroduplexes (Borts and Haber 1987; Waldman and Liskay 1988; Datta et al. 1997; Opperman et al. 2004; Emmanuel et al. 2006). Because the distribution of COR lengths is apparently exponential, it seems that the probability of CO depends on the degree of interparental similarity at a scale which is substantially longer than the characteristic length of a COR (i.e., >100 nt), instead of narrowly localized similarities. Despite the absence of evidences for CO association with conservative regions, COR length distribution in yeast (data from Mancera et al. 2008) do not fit an exponential distribution (supplementary fig. S2, Supplementary Material online, *P* value < 2.2 × 10^−16^, Kolmogorov–Smirnov test).

If preferential occurrence of CO in regions of high interparental similarity is due to MEPS, we expect to see a linear increase of recombination rate with the length of a perfect identity tract. Thus, the distribution of CORs lengths should represent a product of an exponential distribution of intervals between interparental differences yet a linear distribution of COR lengths. This is inconsistent with the exponential distribution of COR lengths that we observed (fig. 1*B*). Thus, the MEPS model is not consistent with our observations, and instead MMR may be a leading factor responsible for association of CO with regions of high interparental similarity.

In yeasts about 600 perfectly identical nucleotides are needed for successful recombination in MMR-competent genotypes, but only about 20 in a MMR deficient genotype (Datta et al. 1997). As most of our CORs are shorter than 35 nt and because high level of heterozygosity may interfere with reparation via homologous recombination due to action of MMR (Stephan and Langley 1992), one might suspect that MMR may be defective in *S*. *commune*. However, core MMR proteins (Msh2, Msh3, Msh6) are all present in *S*. *commune*. Thus, it is more likely that the MMR system in *S*. *commune* is modified to be more tolerant to differences between the recombining genotypes.

In our system recombination between homologous sequences with a high level of nucleotide diversity is not prohibited. This predicts a potentially huge, and possibly disastrous, rate of ectopic recombination. However, if DSB formation occurs only after chromosome pairing, as in *D*. *melanogaster* and *Caenorhabditis elegans* (Page and Hawley 2003), this may reduce the rate of ectopic recombination. Unfortunately, there is no data for *S*. *commune* what comes first, DSB formation or chromosome paring.

### Negative Selection Provides Opportunity for CO

In contrast to our data, several analyses of association between recombination rate and the level of within-population polymorphism all reported positive correlations (Kim et al. 2007; Cutter and Moses 2011; Comeron et al. 2012; Mackay et al. 2012). There are at least two possible explanations for this correlation. First, CO reduces the negative effect of selection, on the level of polymorphism at adjacent sites (Begun and Aquadro 1992; Hudson and Kaplan 1995; Charlesworth 1996). Second, CO may be mutagenic (Lercher and Hurst 2002).

Still, *S*. *commune* is the first hypervariable species in which CO has been studied in close detail. It is likely that patterns of CO in such species are governed by different forces than in species with common, low levels of variation. In particular, because interparental differences are likely to present a stronger obstacle to CO in hypervariable species, the negative correlation between CO and nucleotide diversity is likely to be confined to such species. Also, studies on species with low nucleotide diversity focused on correlations observed between CO and nucleotide diversity within relatively long genome regions. We observed a negative correlation only between CO and nucleotide diversity at a finer scale (<700 nt, fig. 4).

In humans and in *D*. *melanogaster* CO correlates positively with MAF at the kb scale, which has been primarily explained by reduced linkage between neutral variants and alleles under selection (Andolfatto 2001; Lohmueller et al. 2011). Here, we find an opposite trend (fig. 5). Associations of CO with low parental divergence and *π*, reduced MAF, and exon enrichment (figs. 1 and 3–5) all indicate that CO preferentially occurs in genome regions under negative selection. These data suggest that either negative selection on the gene products or on the hotspots themselves may lead to an association of CO with reduced genetic variation in *S*. *commune*.

Could disruption of CO by interparental differences (Waldman and Liskay 1988; Datta et al. 1997; Emmanuel et al. 2006) cause negative selection against new mutations? To test this possibility, we compared MAFs at synonymous sites, the most functionally neutral class of sites in *S*. *commune* (Baranova MA et al. in preparation), in CORs versus 10-kb windows centered at CORs. MAFs were specifically reduced within CORs in both Russian and US populations. Moreover, the US population does not have a significantly lower MAF within the COR for nonsynonymous or all sites, probably due to interplay with other types of selection. In the Russian population MAFs are significantly lower only for all types of sites, probably due to the higher amount of data.

### Biased Gene Conversion Is an Unlikely Explanation of the Observed Patterns

Biased gene conversion (BGC) also may decrease MAFs and *π* (Nagylaki 1984). However, in yeasts the mean conversion tract length is 2 kb (Mancera et al. 2008) whereas the reduction in divergence between the *S*. *commune* parental sequences is mostly confined to the COR. BGC acts on A/T↔G/C SNPs (Nagylaki 1984; Meunier and Duret 2004), but the proportion of these SNPs is equal in CORs and 10-kb genome regions centered at COR (data not shown). Therefore, the reduced within-population diversity in CORs unlikely be the product of BGC but instead is preserved by selection.

### Gene Conversion

Recombination involves formation of heteroduplex, DNA regions that contain two strands from different molecules. If a heteroduplex has mismatches, it can be repaired by the MMR system (Duret and Galtier 2009). MMR degrades a strand that has nicks and resynthesizes it using the intact strand as a template. Thus, conversion tracts are mostly simple (all tracts originate from one genotype): 89% of conversion tracts in yeasts (Mancera et al. 2008) and more than 99% of conversion tracts in humans are simple (Webb et al. 2008). A simple tract is reflected in data just as a single act of switching between parental markers. Therefore, we are unable to estimate length of simple conversion tracts (Webb et al. 2008 encountered the same limitation) without tetrad analysis. Other repair systems may cause complex conversion tracts, where different mismatches may be repaired using different chromosomes as a template. Conversion in humans occurred between homologues with >92% (usually with >95%) identity (Reiter et al. 1998). *Schizophyllum commune* represents a good opportunity to detect complex conversion tracts due to high level of parental sequence divergence, but we detected only one. Failure to detect more may be due to problems with strand invasion exacerbated by extreme differences between the genotypes. Moreover, the observed complex conversion tract is represented only by single marker inherited from the Moscow parent, within a region inherited from the Florida parent, with a nontrivial probability that this marker is the product of the de novo mutation (supplementary fig. S1, Supplementary Material online).

### Recombinational Motifs

The motif we observed in CORs (CNN)n has remote resemblance to other cytosine-rich hotspot elements such as the CCNCCNTNNCCNC motif of Prdm9 in European human (Baudat et al. 2010). In yeast the (CCGNN)_12_ motif has been reported to be associated with transcription and recombination (Kirkpatrick et al. 1999), but no motifs were detected in a more recent genome-wide study (Mancera et al. 2008).

### COs in *S. commune* Occur in Recombinational Hotspots

LD obtained from population data can be a proxy for CO rate (International HapMap 2005; Kim et al. 2007; Myers et al. 2005), with lower LD values indicating higher CO rates (Webb et al. 2008). Thus, reduced LD between SNPs that are separated by a CO (fig. 7) is likely due to COs in *S*. *commune* occurring mostly at recombination hotspots, whose location throughout the *S*. *commune* genome is also clustered by the additional requirement for high sequence similarity. The existence of recombinational hotspots is in agreement with observations in human and yeasts: ≈2% of most active CO sites correspond to ≈30% of events in human (Kong et al. 2008) and 10% of the most active sites in yeasts correspond to more than 50% of all recombination events (Pan et al. 2011). Unfortunately, we do not have enough data to estimate the activity of observed CORs in *S*. *commune*.

Selection also influences LD and regions under positive or negative selection possess elevated LD relative to regions evolving neutrally, because removal of variants in a directed way increases LD (Kiezun et al. 2013). Despite COs in *S*. *commune* preferentially occurring in regions under selection, we observed an association of CO with reduced LD, implying that the effect of stronger selection is not enough to mask the direct effect of recombination on LD.

## Materials and Methods

### DNA Sequencing and Assembly

Spores were isolated from *S*. *commune* fruiting bodies collected from Russia (Moscow) and the Unites States (The Everglades, Florida). The parental strains were haploids initiated as single spore isolates, and the two parental strains were crossed to form a dikaryotic parental genotype. This strain was fruited and single spore derived progeny isolated to obtain the F_1_. We did not collect all four products of meiosis due the complexity of this procedure in *S*. *commune*.

The F_1_ progeny and parents were grown in a liquid minimal medium using 2% ethanol as the carbon source (Raper and Hoffman 1974). DNA was extracted from dried mycelia of parental strains and F_1_ using a CTAB method (Doyle JJ and Doyle JL 1987). Library preparation was performed using TruSeq DNA sample prep kit (Illumina, USA).

We sequenced 17 F_1_ and haploid parental genotypes using an Illumina HiSeq 2000 with paired-end reads of length 101. We trimmed reads with ngShoRT, then independently assembled each genome with soapdenovo (Li et al. 2010) and performed a multiple alignment of all F_1_ offspring, both parental, and reference genomes by MultiZ (Blanchette et al. 2004).

### COs Identification and Validation

We identified all single nucleotide differences between the parents, and using multiple alignments we obtained their coordinates on the reference genome. In all analyses we considered only sites that contained, in both parents, only A, G, C, or T symbols. In the next step, we found all cases among F_1_ where at least 10, 50, or 100 adjacent sites polymorphic between the parents (i.e., interparental differences) matched one parent followed by 10, 50, or 100 sites, respectively, that matched with the other parent.

Using this procedure, we identified 4,707, 1,573, and 671 possible crossovers for 10, 50, and 100 SNPs thresholds correspondently; however, most of them were false positives and lay on contigs boundaries or were associated with misalignments. To exclude false CO events, for each of the possible COs, we tested the alignment using BLAST, excluding local misalignments or regions that had more than one hit and considered only COs that were confirmed by paired-end read mapping, that is, to confirm COs with borders on different contigs we looked for reads or paired reads, that bridged between the contigs. This procedure did not confirm any CORs with interparental differences on different contigs. After alignment and assembly quality control, we retained only 71 COs, which are very unlikely to be the products of any errors.

### Experimental Validation of Crossover Events

To test the correctness of our approach of CO event identification we randomly selected 24 CO regions in 15 F_1_ and sequenced them using Sanger technology. Based on alignment of the F_1_ genome and parental genomes, we designed primers that amplify short (300–600 bp) regions containing putative COs. Primer sequences are listed in the supplementary table S4, Supplementary Material online. Polymerase chain reaction (PCR) was run on a MJ Mini thermal cycler (MJ Research, USA) using the following program: Initial denaturation –95 °C for 3 min, then 95 °C for 15 s, 61C for 30 s, and 72 °C for 30 s, 35 cycles in total. The PCR products were purified using DNA Cleanup Standard kit (Evrogen, Russia) and sequenced using ABI PRISM BigDye Terminator v. 3.1 on an Applied Biosystems 3730 DNA Analyzer (Life Technologies, USA). The resulting sequences completely matched with the assembled genomes, confirming all 24 COs.

### Exon Enrichment

To test whether the observed numbers of CORs that reside within exons or introns were different from a random expectation, we generated a null distribution using 71 randomly chosen regions throughout the genome with the same length of interparental identity tracts (i.e., distance between adjacent polymorphic sites) as observed CORs length. We repeated this procedure 1,000 times to obtain a null distribution.

### Population Patterns

To estimate population genetic parameters, we created an alignment of 33 *S*. *commune* individuals including the reference genome (Ohm et al. 2011), 13 individuals from the United States and 19 individuals from European Russia. To calculate polymorphism levels near CORs or to estimate MAF, we excluded sites not containing at least ten-aligned individuals both from Russian and US populations (parental genomes were not included), if more individuals were aligned at the region of interest, we randomly chose ten of them. To estimate 5% confidence intervals for MAF mean we resampling SNPs of appropriate frequency with the probabilities calculated from observed data. We repeated this procedure 1,000 times to obtain a distribution.

To calculate LD between two sites we considered only pairs of sites that contained biallelic SNPs with at least ten individuals containing nucleotides (no gaps allowed) at both sites. If there were more than ten individuals we randomly sampled ten of them to have equal sample sizes for each SNP pair. Suppose that at the first site there are two alleles *A* and *a* and two alleles at a second site are *B* and *b*. We calculated LD for such sites as:

$$\text{LD}=({\left(x_{\text{11}}-p_{1}q_{\text{1}}\right)}^{\text{2}})/(p_{\text{1}}p_{\text{2}}q_{\text{1}}q_{\text{2}}),$$

where *x*_11_ = *AB* frequency, *p*_1_ = *A* frequency, *p*_2_ = *a* frequency, *q*_1_ = *B* frequency, *q*_2_ = *b* frequency.

### Motif Enrichment

To find any motifs associated with Cos, we used the online version of MEME (Bailey and Elkan 1994) and searched for 1 or 0 motifs per sequence. As input we used COR sequences or COR sequences together with 400-nt flanks. To control for nucleotide content we also tested shuffled sequences and did not find any motifs under such conditions. We also searched for motifs in control sequences of the same length that were adjacent to CORs together with 400 flanking nucleotides and found CNTCNTNNTCNNNCT motif (*e* value 2.0 × 10^−^^15^); however, it was radically less significant than the motif associated with CORs with 400 flanking nucleotides. We also searched for motifs in CORs together with 400 flanking nucleotides using adjacent sequences or random exons as a negative control and discovered a (CNN) _6_ motif in both cases (*e* value 8.2 × 10^−^^42^ and *e* value 9.9 × 10^−^^34^ correspondently).

## Accession Codes

Referenced accessions

Sequence Read Archive

PRJNA234274 and PRJNA236351

## Supplementary Material

Supplementary data S1, tables S1–S5, and figures S1 and S2 are available at *Molecular Biology and Evolution* online (http://www.mbe.oxfordjournals.org/).

Supplementary Data
