# Supplementary material for: Crossing-Over in a Hypervariable Species Preferentially Occurs in Regions of High Local Similarity
Source: Mol Biol Evol. 2014 Aug 18;31(11):3016–25. doi: 10.1093/molbev/msu242 (PMC4209137; doi:10.1093/molbev/msu242)
Supplement: Supplementary Data [file supp_31_11_3016__index.html]

Crossing-Over in a Hypervariable Species Preferentially Occurs in Regions of High Local Similarity — Crossing-Over in a Hypervariable Species Preferentially Occurs in Regions of High Local Similarity — Supplementary Data 

# Crossing-Over in a Hypervariable Species Preferentially Occurs in Regions of High Local Similarity

## Supplementary Data

files

**Files in this Data Supplement:**

- Supplementary Data - doc file
