# Supplementary material for: Crossing-Over in a Hypervariable Species Preferentially Occurs in Regions of High Local Similarity
Source: Mol Biol Evol. 2014 Aug 18;31(11):3016–25. doi: 10.1093/molbev/msu242 (PMC4209137; doi:10.1093/molbev/msu242)
Supplement: Supplementary Data [file supp_msu242_suppl.doc]

**Supplementary Figures**

**Figure S. 1. Complex conversion tract.** Red arrow marks interparental differences, where offspring allele matched with variant in Moscow parent among region inherited from The Everglades parent.

**Figure S. 2. Distribution of CORs lengths in yeast fitted by exponential distribution. COR length distribution estimated using data obtained from Mancera et al. 2008.**

**Table S. 1.** Assembly and coverage data for F_1_ offspring and parental strains.

| **Lineage** | **Length of all contigs** | **Avarege length of contigs** | **Longest scaffold** | **N50** | **Coverage** |
| --- | --- | --- | --- | --- | --- |
| **F1-6** | 35668223 | 15480 | 242739 | 40336 | 33.2 |
| **F1-2** | 37432709 | 15198 | 315559 | 45209 | 44.0 |
| **Moscow** | 32002501 | 16453 | 244881 | 45849 | 66.2 |
| **F1-5** | 34467377 | 17775 | 349553 | 47741 | 42.8 |
| **F1-12** | 34316109 | 18469 | 367359 | 55718 | 93.8 |
| **F1-26** | 33504275 | 19708 | 450797 | 65043 | 68.3 |
| **F1-30** | 32158627 | 20614 | 624909 | 65188 | 86.6 |
| **F1-22** | 33204124 | 22165 | 408258 | 69177 | 76.7 |
| **F1-29** | 33999060 | 22471 | 403319 | 71490 | 71.0 |
| **F1-25** | 33251428 | 22604 | 422115 | 71526 | 98.4 |
| **F1-3** | 34003590 | 23147 | 411798 | 73445 | 63.4 |
| **F1-19** | 35479722 | 19732 | 477758 | 74290 | 56.8 |
| **F1-18** | 34462529 | 23816 | 474004 | 75151 | 109.6 |
| **Everglades** | 33566331 | 22332 | 436143 | 75823 | 53.0 |
| **F1-17** | 32914583 | 22239 | 423109 | 79634 | 115.8 |
| **F1-16** | 35280030 | 23379 | 440228 | 85007 | 124.6 |
| **F1-9** | 34501547 | 25183 | 580735 | 89724 | 96.6 |
| **F1-10** | 35040845 | 24957 | 554590 | 97532 | 114.4 |
| **F1-14** | 35212230 | 25026 | 627564 | 105558 | 114.5 |

**Table S. 2.** Assembly and coverage data for unrelated individuals.

| **Lineage** | **Length of all contigs** | **Avarege length of contigs** | **longest scaffold** | **N50** | **Coverege** |
| --- | --- | --- | --- | --- | --- |
| **A10** | 34320592 | 16717 | 267062 | 45565 | 55.39542 |
| **A13** | 32385397 | 13578 | 156860 | 23905 | 62.24979 |
| **A3** | 33698200 | 25704 | 548204 | 99862 | 86.98884 |
| **A4** | 27949639 | 13891 | 185121 | 35638 | 52.80354 |
| **A5** | 34222472 | 20083 | 390609 | 71563 | 33.97784 |
| **A7** | 16526250 | 7111 | 98261 | 15212 | 37.65457 |
| **A8** | 33976189 | 25111 | 504461 | 115436 | 77.66482 |
| **B1** | 33409855 | 24969 | 557278 | 91799 | 93.07908 |
| **B2** | 50494874 | 3257 | 213179 | 5326 | 91.60616 |
| **B3** | 33925609 | 25074 | 549807 | 109182 | 74.68085 |
| **B5** | 20609865 | 3915 | 35783 | 4990 | 49.72629 |
| **B6** | 12679944 | 3155 | 36292 | 4328 | 31.43473 |
| **K2** | 39045765 | 2396 | 168747 | 3090 | 94.32408 |
| **K3** | 30062873 | 22809 | 233234 | 56690 | 232.6906 |
| **K4** | 30794970 | 20101 | 225654 | 49767 | 92.238 |
| **M1** | 32588010 | 21567 | 280651 | 51598 | 84.22507 |
| **M2** | 33027381 | 21076 | 207326 | 47442 | 77.65692 |
| **M3** | 32785103 | 20895 | 214528 | 54538 | 87.06902 |
| **M4** | 18255284 | 11954 | 131268 | 25993 | 60.96019 |
| **M5** | 31711463 | 20198 | 276727 | 50648 | 82.6998 |
| **M6** | 31129054 | 20425 | 353709 | 57049 | 96.67665 |
| **M7** | 33070164 | 16274 | 289745 | 48526 | 95.61147 |
| **M8** | 36792526 | 3169 | 186300 | 4687 | 85.3575 |
| **Mi1** | 33558961 | 27826 | 724145 | 113818 | 109.6452 |
| **P1** | 32649521 | 20431 | 242967 | 47532 | 48.92049 |
| **P3** | 38102880 | 22256 | 372527 | 52475 | 130.1263 |
| **S1** | 17264627 | 7061 | 69366 | 11754 | 53.68486 |
| **S2** | 32394235 | 23440 | 364632 | 64226 | 108.889 |
| **S4** | 16400753 | 9921 | 147027 | 19311 | 96.03329 |
| **S5** | 33939623 | 10278 | 209882 | 33550 | 75.11443 |

**Table S. 3**. Reference coordinates of CORs. Asterisk depicts event with conversion tract.

| **Reference scaffold** | **start** | **end** | **CO region** |
| --- | --- | --- | --- |
| **Ref.scaffold_1** | 53246 | 53259 | 5 |
| **Ref.scaffold_1** | 2089708 | 2089718 | 6 |
| **Ref.scaffold_1** | 2391753 | 2391833 | 7 |
| **Ref.scaffold_1** | 2494757 | 2494812 | 8 |
| **Ref.scaffold_1** | 2737771 | 2737801 | 9 |
| **Ref.scaffold_1** | 3906988 | 3907069 | 44 |
| **Ref.scaffold_1** | 3911123 | 3911150 | 10 |
| **Ref.scaffold_1** | 4423174 | 4423183 | 36 |
| **Ref.scaffold_1** | 4895597 | 4895646 | 11 |
| **Ref.scaffold_10** | 16367 | 16382 | 62 |
| **Ref.scaffold_10** | 70087 | 70149 | 37 |
| **Ref.scaffold_10** | 88568 | 88581 | 25 |
| **Ref.scaffold_10** | 1655680 | 1655726 | 46 |
| **Ref.scaffold_11** | 354469 | 354517 | 21 |
| **Ref.scaffold_11** | 678137 | 678185 | 22 |
| **Ref.scaffold_11** | 1080770 | 1080832 | 45 |
| **Ref.scaffold_11** | 1094343 | 1094400 | 60 |
| **Ref.scaffold_12** | 57136 | 57168 | 61 |
| **Ref.scaffold_12** | 528561 | 528582 | 54 |
| **Ref.scaffold_13** | 231448 | 231469 | 31 |
| **Ref.scaffold_13** | 447060 | 447075 | 41 |
| **Ref.scaffold_15** | 585978 | 586155 | 59 |
| **Ref.scaffold_16** | 147379 | 147729 | 27 |
| **Ref.scaffold_16** | 571639 | 571707 | 35 |
| **Ref.scaffold_19** | 272354 | 272450 | 38 |
| **Ref.scaffold_2** | 340959 | 341086 | 50 |
| **Ref.scaffold_2** | 490890 | 490899 | 17 |
| **Ref.scaffold_2** | 1304627 | 1304796 | 18 |
| **Ref.scaffold_2** | 1477858 | 1477955 | 32 |
| **Ref.scaffold_2** | 1880991 | 1881030 | 19 |
| **Ref.scaffold_2** | 1890155 | 1890206 | 20 |
| **Ref.scaffold_2** | 2282900 | 2283052 | 49 |
| **Ref.scaffold_2** | 2484752 | 2484781 | 48 |
| **Ref.scaffold_2** | 3064386 | 3064482 | 53 |
| **Ref.scaffold_2** | 3202775 | 3202793 | 56 |
| **Ref.scaffold_2** | 3509746 | 3509758 | 39 |
| **Ref.scaffold_25** | 28933 | 28966 | 26 |
| **Ref.scaffold_3** | 1109707 | 1109737 | 55 |
| **Ref.scaffold_3** | 1280179 | 1280185 | 64 |
| **Ref.scaffold_3** | 1860038 | 1860107 | 42 |
| **Ref.scaffold_3** | 2371923 | 2371938 | 1 |
| **Ref.scaffold_3** | 2404267 | 2404277 | 2 |
| **Ref.scaffold_3** | 2547204 | 2547286 | 3 |
| **Ref.scaffold_3** | 2661282 | 2661291 | 4 |
| **Ref.scaffold_4** | 439915 | 439927 | 12 |
| **Ref.scaffold_4** | 461334 | 461472 | 13 |
| **Ref.scaffold_4** | 2215462 | 2215475 | 34 |
| **Ref.scaffold_4** | 3005889 | 3005943 | 14 |
| **Ref.scaffold_5** | 1475514 | 1475585 | 15 |
| **Ref.scaffold_5** | 1706836 | 1706858 | 40 |
| **Ref.scaffold_5** | 2185906 | 2185910 | 51 |
| **Ref.scaffold_5** | 2218878 | 2218919 | 16 |
| **Ref.scaffold_6** | 853849 | 853945 | 47 |
| **Ref.scaffold_6** | 1796726 | 1796843 | 58 |
| **Ref.scaffold_6** | 1845968 | 1845985 | 57 |
| **Ref.scaffold_7** | 1450476 | 1450687 | 23 |
| **Ref.scaffold_8** | 1060512 | 1060523 | 28 |
| **Ref.scaffold_8** | 1082936 | 1082960 | 29 |
| **Ref.scaffold_8** | 1577117 | 1577196 | 33 |
| **Ref.scaffold_8** | 1869611 | 1869699 | 30 |
| **Ref.scaffold_8** | 1885819 | 1885837 | 63 |
| **Ref.scaffold_9** | 717245 | 717290 | 43 |
| **Ref.scaffold_9** | 981731 | 981747 | 52 |
| **Ref.scaffold_9** | 1244161 | 1244179 | 24 |
| ***Ref.scaffold_1** | 4902130 | 4902193 | 70 |
| **Ref.scaffold_4** | 2602316 | 2602335 | 69 |
| **Ref.scaffold_4** | 1321023 | 1321064 | 66 |
| **Ref.scaffold_4** | 2257051 | 2257078 | 67 |
| **Ref.scaffold_5** | 1965249 | 1965295 | 71 |
| **Ref.scaffold_5** | 237731 | 237740 | 65 |
| **Ref.scaffold_12** | 119982 | 120014 | 68 |

**Table. S. 4**. Primers used for CO validation.

| **Primer name** | **Sequence (5’-3’)** | **Length** | **CO region in suppl. data** |
| --- | --- | --- | --- |
| **F1_10.scaffold927_L** | GACGACGAGTATGGCGACCTGCTC | 24 | **32** |
| **F1_10.scaffold927_R** | GGCTGAGGCGCGAAAACGACA | 21 |
| **F1_12.scaffold75768169618+0_F** | CTCCCTGGAGGTCTTCGCGG | 20 | **26** |
| **F1_12.scaffold757_R** | ATCGCTAGGTTGAGGTTCACGTCGG | 25 |
| **F1_12.scaffold811_F** | TCCGGACCACACACGAAGTACTG | 23 | **16** |
| **F1_12.scaffold811_R** | CCTCCTGCACAAGCACAAGCA | 21 |
| **F1_16.scaffold1381_F** | CTTCAGGAGCTTCGCGTCCGC | 21 | **22** |
| **F1_16.scaffold1381_R** | TGACGCGCGCCATTGTTCTCGA | 22 |
| **F1_16.scaffold237_F** | GAAGGACAAGTCGGCGAACAAGG | 23 | **14** |
| **F1_16.scaffold237_R** | TGAGTGCATGCTGGACATCGC | 21 |
| **F1_16.scaffold511_F** | CTAAGGAGCTTGGGAAGGAGGAGCTA | 26 | **19** |
| **F1_16.scaffold511_R** | AGGGCTTTGCCATGCACTGC | 20 |
| **F1_17.C47277_F** | ATATTTCTGACGGCGTTCCCTTCGA | 25 | **1** |
| **F1_17.C47277_R** | AAACCGGTGCAATCCTCTGACCAC | 24 |
| **F1_19.scaffold1225_F** | GCCCTTCCAGTACTTGCAGACAA | 23 | **5** |
| **F1_19.scaffold1225_R** | CGATATGCTCGCCACGACGAC | 21 |
| **F1_19.scaffold894_F** | CCGCGCAAAAGTTCAACTTCGTCACA | 26 | **28** |
| **F1_19.scaffold894_R** | AAGACGCCGGCGGTGTTGTATGT | 23 |
| **F1_2.scaffold1821_F** | GCCACTGGTGTCAACAATGGTGG | 23 | **13** |
| **F1_2.scaffold1821_R** | GCATGGGCATGTAGGCATAGCA | 22 |
| **F1_22.scaffold1051_F** | GGAAAGAGGAGCTGTCAACTGCGAA | 25 | **17** |
| **F1_22.scaffold1051_R** | GAAGGCGGCTTCAGTTGAGGTCAT | 24 |
| **F1_22.scaffold1236_F** | CGACAAGCTCGTCTGTGCGTCACA | 24 | **29** |
| **F1_22.scaffold1236_R** | CGACCGCATTCGCCTTCGTTCC | 22 |
| **F1_25.scaffold119_F** | ACATGGACATCAAGCCGCACAA | 22 | **15** |
| **F1_25.scaffold119_R** | CGCTTGTTCCCGCTGATAATCTGC | 24 |
| **F1_25.scaffold757_F** | AGTAGACGCGATGTTCCTGGGCT | 23 | **18** |
| **F1_25.scaffold7578_R** | GTCCCGTATGCACTCACGCGTC | 22 |
| **F1_26.scaffold1314_F** | GTGCGTGAGATCCACCCAAGG | 21 | **6** |
| **F1_26.scaffold1314_R** | AATTCAAGGGCAGGGCCTTCG | 21 |
| **F1_26.scaffold1615_F** | CGCCAGCCTTTACCTCATTTTCCCG | 25 | **27** |
| **F1_26.scaffold1615_R** | GTGGCGCTCGCTTCCTCTTC | 20 |
| **F1_30.scaffold1359_F** | AGACCTAAGGCCGGCAGACA | 23 | **3** |
| **F1_30.scaffold1359_R** | AGACCTAAGGCCGGCAGACA | 20 |
| **F1_30.scaffold315_F** | AGGCGACAATGAGGGTCACCACA | 23 | **30** |
| **F1_30.scaffold315_R** | CGTTCCCAATGGGTGAGCGCAATT | 24 |
| **F1_30.scaffold618_F** | CCGTCGTCTCCGTGACTGAAGG | 22 | **12** |
| **F1_30.scaffold618_R** | GCGAAGATCTTTGGTCCGCGG | 21 |
| **F1_30.scaffold933_F** | CTCCAAATCCCAGCGCGTCGT | 21 | **24** |
| **F1_30.scaffold933_R** | AAACACATCCAACGGCCCATCGTTC | 25 |
| **F1_5.scaffold844_F** | GCTTCTCTGACCTCGACCGTTG | 22 | **4** |
| **F1_5.scaffold844_R** | GAGGAAACACGCGAAGTAGCC | 21 |
| **F1_9.scaffold134_F** | TTACCGAGCATAGCCAACCGCC | 22 | **31** |
| **F1_9.scaffold134_R** | CTTGGGCTGATGTGAGCGCGA | 21 |
| **F1_9.scaffold1360_F** | AACCACCGACTACTGCGCAC | 20 | **7** |
| **F1_9.scaffold1360_R** | TTCCTTTGCTGAGCCTAGCGC | 21 |
| **F1_9.scaffold789_F** | CGCGTCGAGGCCATGGTTGT | 20 | **23** |
| **F1_9.scaffold789_R** | GGAGCCGCCGCAACAGGAG | 19 |

**Table S. 5.** Tajima`s D values for 10 randomly sampled individuals from the Russian population and the USA population for polymorphic loci within CORs and within 10 kb regions centred at CORs suited as control.

| **Class of sites** | **Region** | **Population** | **Tajima`s D value** |
| --- | --- | --- | --- |
| **synonymous** | COR | Russia | -0.19 |
| **synonymous** | COR | USA | -0.86 |
| **non-synonymous** | COR | Russia | -0.55 |
| **non-synonymous** | COR | USA | -0.81 |
| **all sites** | COR | Russia | -0.35 |
| **all sites** | COR | USA | -0.87 |
| **synonymous** | control | Russia | 0.05 |
| **synonymous** | control | USA | -0.53 |
| **non-synonymous** | control | Russia | -0.17 |
| **non-synonymous** | control | USA | -0.78 |
| **all sites** | control | Russia | 0.00 |
| **all sites** | control | USA | -0.66 |
